# Anchored Cu single atoms on porous g-C_3_N_4_ for superior photocatalytic H_2_ evolution from water splitting†

**DOI:** 10.1039/d3ra00775h

**Published:** 2023-03-17

**Authors:** Tong Zhou, Haitang Wei, Bin Xiao, Tianping Lv, Liangfei Duan, Qingjie Lu, Jin Zhang, Yumin Zhang, Qingju Liu

**Affiliations:** a National Center for International Research on Photoelectric and Energy Materials, Yunnan Key Laboratory for Micro/Nano Materials & Technology, School of Materials and Energy, Yunnan University Kunming 650091 P. R. China ynuxb2011@163.com +86-871 65032713

## Abstract

One of the most promising strategies for producing hydrogen is photocatalytic water splitting, in which the photocatalyst is a key component. Among many semiconductor photocatalysts, g-C_3_N_4_ has attracted great attention due to its narrow band gap, excellent stability and low cost. However, practical application is limited by its poor intrinsic activity. In this work, a high-performance porous g-C_3_N_4_ (PCN) photocatalyst with anchored Cu single atoms (CuSAs) was synthesized by a one-step co-heating approach. The obtained Cu1.5–PCN displays an excellent hydrogen evolution rate (HER) of 2142.4 μmol h^−1^ g^−1^ under visible light (=420 nm), which is around 15 and 109 times higher than those of PCN and bulk g-C_3_N_4_, respectively. In addition, it also shows good stability during H_2_ evolution. The results of experimental research and DFT simulations indicate that the single Cu ions formed bonds with the N-ring and these remain stable. Meanwhile, the special electronic structure of the Cu–N charge bridge extends the light absorption band to the visible-light region (380–700 nm). This high-performance and low-cost photocatalyst has great potential in solar energy conversion.

## Abstract 2

Single Cu atom with special structure of Cu–N charge bridge broadens the response range of visible light, increases the efficiency of electron–hole separation and transfer, and reduces the hydrogen evolution reaction energy barrier.

## Graphical abstract

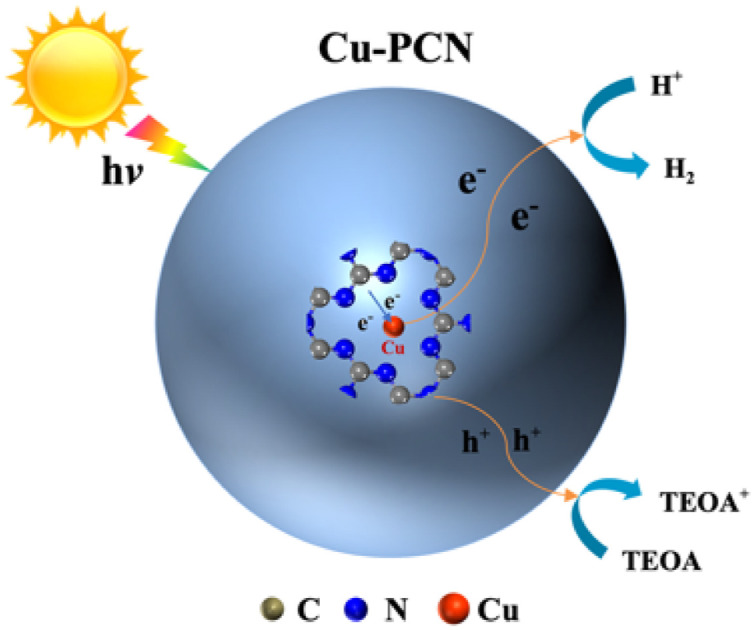

## 1 Introduction

Given the accelerated economic growth and energy demand, hydrogen is expected to be an important alternative source for clean energy and sustainable development.^1^ One of the most effective ways to turn solar energy into H_2_ energy is photocatalytic water splitting, in which the photocatalyst is a key component.^2^ Since the discovery of the photocatalytic effect in 1972, various semiconductor photocatalysts have been extensively studied and serial progresses have been made.^3–5^ Among those photocatalysts, g-C_3_N_4_ has received a lot of interest due to its visible-light responsiveness, high stability, non-toxicity and low cost, *etc.*^6–8^ However, the photocatalytic activity of pristine bulk g-C_3_N_4_ (BCN) is quite low owing to the fast charge recombination and low specific surface area (SSA),^9^ which greatly hinders its industrial application. To improve the photocatalytic activity, numerous research on the modification of BCN has been carried out, including ion doping,^10^ morphology controlling,^11,12^ hydrogenated,^13^ heterostructure constructing,^14–17^ magnetic-field-promoted photocatalytic,^18^ precious metal loading,^19^*etc.* Recently, single-atom catalysts (SACs) for photocatalytic H_2_ evolution have drawn tremendous attention for the maximum utilization of atoms and high efficiency of photo-generated charge separation.^20,21^ Xiang *et al.*^22^ reported that Cu single atoms (CuSA) were loaded on crystalline CN nanorod by molten salts and the reflux method, and the photocatalytic activity of the CuSA loaded sample enhanced nearly 2 times. However, it was difficult to anchor large amounts of single atoms due to the low specific surface area of CN. Zou *et al.*^23^ modified the configuration of CN and Ag single atoms to form Ag–N_2_C_2_/CN to increase the metal loading amount, and the H_2_ evolution rate of the as-prepared Ag–N_2_C_2_/CN enhanced almost 4 times than that of Ag–CN. Guo *et al.*^24^ loaded Pt single atoms on the CN with N vacancies, and Pt–C bond was formed. Due to the small SSA (49.7 m^2^ g^−1^) and low efficient charge transfer between CN and Pt, the highest AQE is 0.544% at 420 nm. Zhang *et al.*^25^ analyzed charge carrier transmission in transition-metal single atoms loaded with CN by first-principles theory, and the results indicate that only Cu could form Cu–N bond on the surface of CN which could accelerate the photo-generated charges migration.

From these works, it can be concluded that the configuration and morphologies of the CN substrate and the chemical bonding between CN and metal single atoms (M–C or M–N, M = metal) are most essential to form high-efficiency and high-stable M–CN photocatalysts. Moreover, the 3d transition metal Cu not only has different oxidation states but also owns a cheaper price than precious metals, which is one of the best cocatalysts for improving the activity and reducing the cost of CN. In contrast to BCN, ultrathin (2D-like) and porous g-C_3_N_4_ (PCN) will be better substrates for loading single atoms, which is attributable to the cognate morphology of graphene.^26^ The porous structure of PCN exposes more surface which is favorable to the high loading amount and uniform dispersion of metal.^27^

In this work, we present a one-step co-heating solution approach to synthesize porous g-C_3_N_4_ and anchor CuSA on its surface, and the visible-light-responsive Cu1.5–PCN photocatalyst with rich pores and high SSA is obtained, where the CuSA does not aggregate even in the high-temperature process. The synthesized catalysts exhibit high stability and excellent photocatalytic H_2_ evolution performance (apparent quantum yields: 23.7%, *λ* = 400 nm; 19.3%, *λ* = 420 nm) in the presence of triethanolamine as hole scavenger under simulated sunlight, without any precious metals as co-catalyst. Furthermore, the mechanism of the high activity is studied through both experimental characterization and theoretical calculation, and a reaction mechanism leading to the improved photocatalytic performance of Cu1.5–PCN photocatalysts is proposed.

## 2 Experimental

### 2.1 Materials

All chemical reagents in this work were used without further purification. Ultrapure water was used in all the experiments. Copper nitrate trihydrate (Cu(NO_3_)_2_·3H_2_O, 98%), urea (99%), sodium sulfate anhydrous (Na_2_SO_4_, 99%), and triethanolamine (TEOA, 97%) were purchased from Aladdin Industrial Incorporation.

### 2.2 Preparation of g-C_3_N_4_ and Cu-doped g-C_3_N_4_

The CuSA anchored on porous g-C_3_N_4_ (denoted as Cu1.5–PCN) photocatalysts were synthesized by the following steps: firstly, urea (2 g) was dissolved in ultrapure water by ultrasonic to form a homogeneous solution. Secondly, Cu (NO_3_)_2_·3H_2_O (6.59 mg) was added while the solution was constantly stirred. Then the mixed solution was sonicated at room temperature for several minutes to further disperse uniformly the solution. Finally, the resultant mixture solution was put into a muffle furnace and calcined after being placed in a covered corundum crucible at 400 °C for 1 h and then to 500 °C for 2 h in air atmosphere with a heating rate of 10 °C min^−1^. The products were gathered after cooling to room temperature and ground to obtain Cu1.5–PCN with a 1.5 wt% Cu content. By the method, a series of Cu–PCN catalysts with different Cu loading amounts were prepared by changing the Cu (NO_3_)_2_·3H_2_O amount.

As the controlled sample, bulk g-C_3_N_4_ (BCN) was synthesized *via* calcination of urea at 400 °C for 1 h and then to 500 °C for 2 h in air atmosphere with 10 °C min^−1^. The synthesis procedure of the porous g-C_3_N_4_ (PCN) was similar to that of Cu1.5–PCN, except for not adding Cu(NO_3_)_2_·3H_2_O precursor.

## 3 Results and discussion

**Fig. 1:**
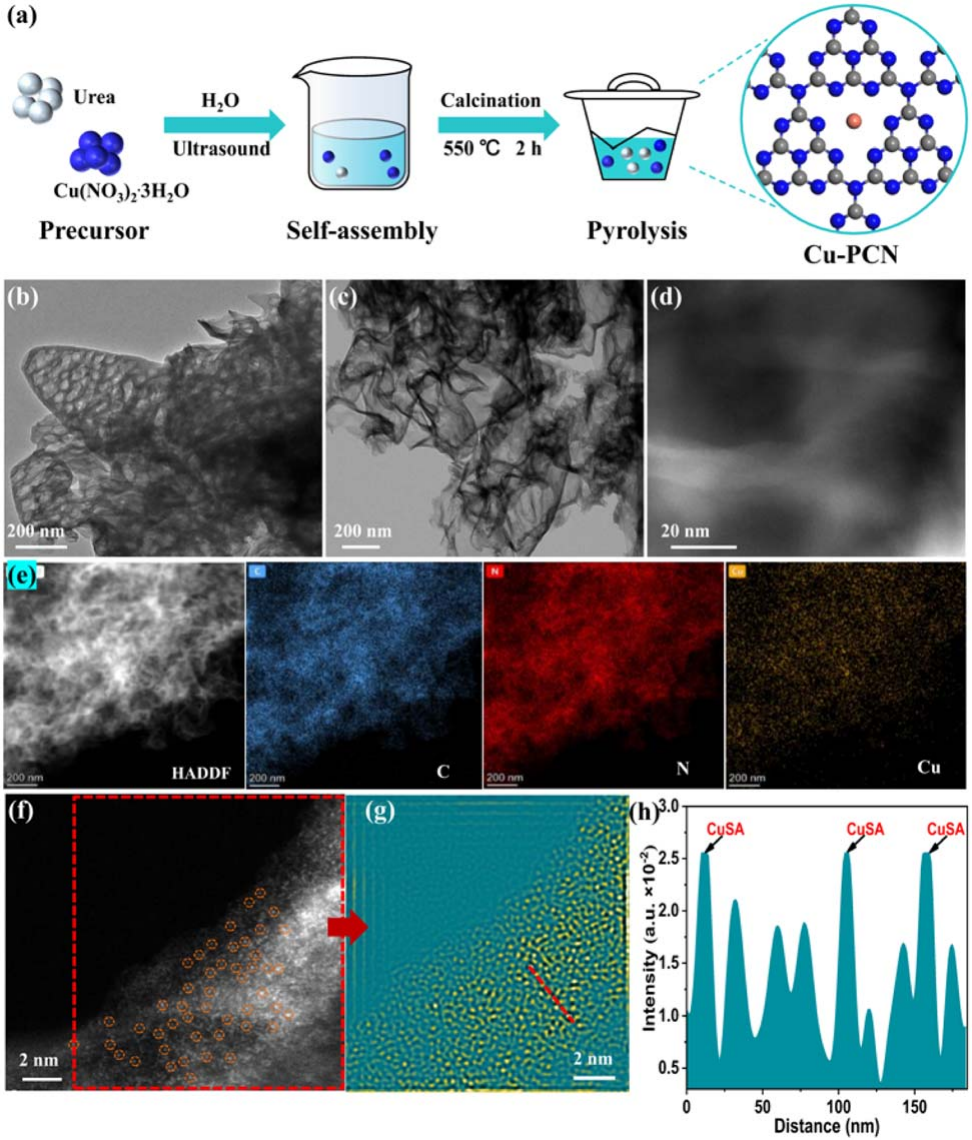
(a) Synthetic route for the preparation of Cu1.5–PCN photocatalysts; (b and c) TEM images of PCN and Cu1.5–PCN, respectively; (d) HAADF-STEM images of Cu1.5–PCN; (e) HAADF-STEM and EDS elemental; mapping images (scale bar, 200 nm) of Cu1.5–PCN; (f and g) spherical aberration-corrected HAADF-STEM images of Cu1.5–PCN, the bright dots in the orange circles are Cu single atoms and the pseudo color image of the area (f) marked in; (h) the intensity profiles of the lattice fringe corresponding to yellow dotted arrow in (g).

The Cu anchored on porous g-C_3_N_4_ (Cu1.5–PCN) samples were synthesized through a simple one-pot calcination strategy as displayed in Fig. 1a. Compared with the bulk g-C_3_N_4_ (BCN) (Fig. S1†), PCN and Cu1.5–PCN are composed of loosely curled nanosheets as revealed by the SEM images, with morphological characteristics of irregular flocculent petals (Fig. S2†). The TEM images of PCN and Cu1.5–PCN samples as shown in Fig. 1b and c, respectively. The PCN has a porous structure, while Cu1.5–PCN has an ultrathin layered structure (similar to few-layered graphene with pores). We retained the water before calcining the precursor (as seen in Fig. 1a); and a large amount of water-vaper was generated during the high-temperature pyrolysis of the precursor. The burst of the bubbles during the heat treatment process may lead to the formation of nanopores.

As illustrated in Fig. S3,† compared with BCN, which showed peaks at 13.1° (corresponding to the {001} lattice plane diffraction) and 27.4° (corresponding to the {100} lattice plane diffraction), the intensities of the peaks PCN and Cu1.5–PCN are weakened and slightly shifted to higher angles. In particular, Cu1.5–PCN has obvious amorphous characteristics, which is confirmed by the weakening of diffraction peaks for carbon nitride.^28^ The XRD patterns of the other catalysts are shown in Fig. S3.† These results indicate that the successful combination of PCN and single Cu atom, as the layer-to-layer ordering is decreased by single-atoms incorporation.^22^

In addition, large metal nanoparticles with size (>few nanometer) are not observed in the high resolution (HR)-STEM image (Fig. 1d). The element mapping (Fig. 1e and S4†) by energy-dispersive X-ray energy spectra (EDS, Fig. S5†) shows that Cu is successfully loaded on the porous g-C_3_N_4_ matrix, and the C, N, and Cu elements are uniformly distributed throughout the Cu1.5–PCN. As revealed by spherical aberration corrected high-angle dark-field scanning transmission electron microscope (HAADF-STEM) images (Fig. 1f and g and S6†), instead of Cu metal nano-particles, monodisperse isolated Cu atoms were observed (circled in Fig. 1f).

**Fig. 2:**
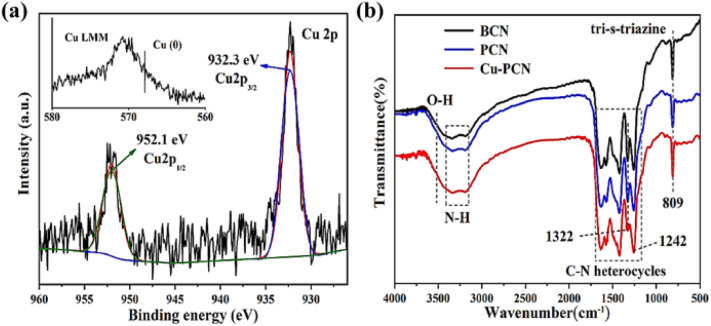
(a) The high-resolution Cu2p XPS spectrum and Cu LMM auger spectrum (inset) of Cu1.5–PCN, (b) FT-IR spectra of BCN, PCN and Cu1.5–PCN.

The chemical composition and valence state of Cu1.5–PCN were investigated through XPS and the Cu single atoms are found to exist as Cu^1+^ ions bonded with N. The high-resolution Cu2p XPS spectrum (Fig. 2a) was deconvoluted into two main peaks at 932.3 eV and 952.1 eV, corresponding to Cu2p_3/2_ and Cu2p_1/2_, respectively. No related shake-up lines are observed in the high-resolution Cu2p spectrum and because of the absence of a satellite structure, the signal could be attributed to Cu^0^ or Cu^1+^. Further combine the Cu LMM auger spectrum to confirm the absence of Cu^0^ state.^29^ The C 1s spectra (Fig. S7†) of Cu1.5–PCN display the peaks centered at 284.8 and 288.1 eV, which corresponding to the sp^2^ C–C bond and sp^2^ bond of hybridized carbon in N–C=N,^36^ respectively. As for the N 1s spectrum of Cu1.5–PCN, as shown in Fig. S7,† the peaks could be divided into four main peaks at 398.3 eV, 399.6 eV, 400.9 eV and 404.0 eV, which could be ascribed to the sp^2^-hybridized aromatic nitrogen atoms connected to carbon atoms (C–N=C), tertiary N atoms (sp^3^ hybridized nitrogen atom (N–(C)_3_)), C–NH_*x*_ groups and possible positively charged nitrogen atoms in CN heterocycles.^30–32^ The C1s and N1s spectrum of Cu1.5–PCN are further compared with the spectra of undoped g-C_3_N_4_ and an insignificant difference is found, which demonstrates that the incorporation of Cu would not affect the basic structural features of carbon nitride during synthesis. It is worth noting that the binding energy of C–N=C (ring N) in the N1s spectrum for Cu1.5–PCN is higher than that of PCN, indicating the formation of Cu–N coordinate bonds. The chemical functional groups of the BCN, PCN and Cu1.5–PCN were measured by FTIR spectra, and the results are shown in Fig. 2b. Particularly, the sharp band at 809 cm^−1^ is ascribed to the characteristic stretching mode of tri-*s*-triazine ring,^33^ the peaks at 1322 and 1242 cm^−1^ are attributed to the C=NH–C (ring N) and aromatic N–(C)_3_ (tertiary N) stretching vibration modes, respectively.^30,34^ It is worth noting that the intensity of Cu1.5–PCN at 1322 cm^−1^ is not obvious in the spectra, while the peak at 1242 cm^−1^ is almost the same as PCN and BCN. Moreover, the FTIR spectra of Cu1.5–PCN with higher Cu loading amount showed similar spectra (Fig. S8†). The above results are consistent with the XPS spectrum, indicating that Cu incorporated does not affect the basic chemical framework of PCN, and the Cu atoms are coordinated with PCN through the ring N sites. Therefore, the monodisperse Cu single atom was successfully introduced to the N-ligand cavity of PCN (in the form of Cu-ions bonded with N).

**Fig. 3:**
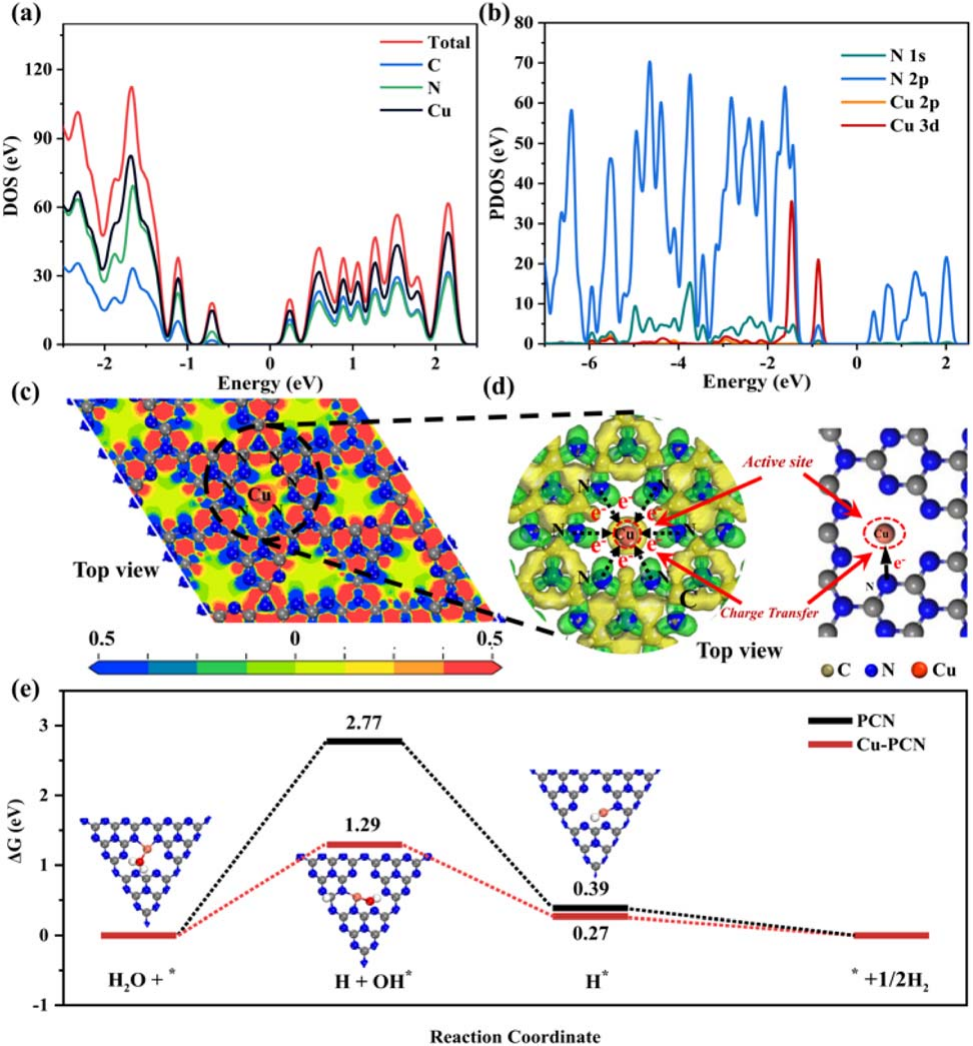
(a) Calculated total DOS of prepared catalysts, (b) the PDOSs of N 1s, N 2p, Cu 2p and Cu 3d orbital distribution, (c) the electronic location function, (d) partial charge density distributions and electron transfer path of catalysts, (e) calculated Gibbs free adsorption energy profiles of HER for the as-synthesized catalysts.

According to the findings of calculations using the density functional theory (DFT), the atom orbitals of Cu and N mainly devote the valence band (VB) in the density of states (DOS), demonstrating that Cu and the surrounding N atoms have distinct restricted electronic effects (Fig. 3a). In addition, we further discussed the orbital hybridization of elements by calculating the projected density of states (PDOS) of Cu and N. As seen in Fig. 3a and b, the DOS of N2p and Cu3d tightly overlapped, indicating that the p-d orbitals of Cu–N atoms are hybridized. Porous g-C_3_N_4_ has well anchored single Cu atoms due to the tight charge interaction between Cu and N. The charge energy diagram around the element (CED, Fig. 3c) also shows that there is a strong charge interaction between Cu and N elements, which means that the N atoms in the porous g-C_3_N_4_ substrate anchor the single Cu atoms. This perhaps attributed initially to the porous of the g-C_3_N_4_, originating from the sp^2^-hybridized aromatic N atoms in the tri-*s*-triazine ring, which tends to anchor, and coordinate monodisperse metal atoms.^35,36^ In addition, the partial electron density distributions indicates that charges will be transferred to the connected N atoms through C atoms, and finally through N atoms to reach Cu atoms (Fig. 3d). Additionally, we further calculated the adsorption Gibbs free energy (ΔGH) of various intermediate products that may be generated for PCN and Cu1.5–PCN during the photocatalytic water splitting process. It is more advantageous for H_2_ production when the energy barrier needed for the H_2_ evolution reaction process is lower. Obviously, after introducing Cu atoms into PCN, the (ΔGH–OH*, ΔGH*) values are (1.29, 0.27) are significantly lower than that of the pure PCN (2.77, 0.39). The results show that the Cu1.5–PCN is more conducive to the dissociation of water and the generation of hydrogen.^37^ The H_2_ evolution process on Cu1.5–PCN is shown in Fig. S9.†

**Fig. 4:**
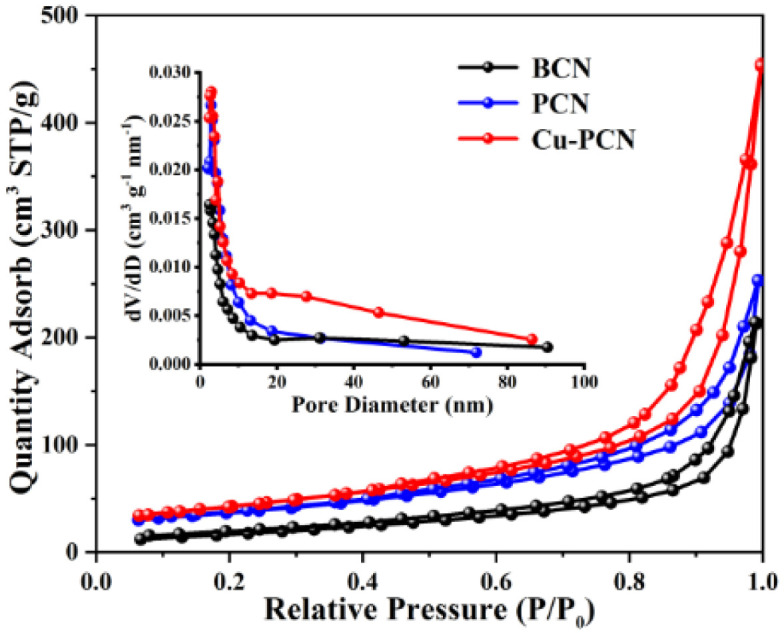
N_2_ adsorption/desorption isotherms and pore diameter distribution plots (inset) of BCN, PCN and Cu1.5–PCN.

The N_2_ adsorption–desorption isotherms of the BCN, PCN and Cu1.5–PCN photocatalysts are shown in Fig. 4. All photocatalysts possess typical type IV isotherms with H3 type hysteresis loop, indicating the predominant mesoporous characteristic, which corresponds to the results of transmission electron microscope. Additionally, the Barret–Joyner–Halenda (BJH) pore-diameter distribution plots are displayed in the inset of Fig. 4. The pore size distributions of the PCN and Cu1.5–PCN photocatalysts present most of the pore diameters between 20 and 30 nm, indicating the presence of mesopores. The obtained BET specific surface area, average pore diameter and pore volume of photocatalysts are shown in Table S1.† Generally, more surface area can effectively increase the contact area, thereby providing more abundance of active sites.^38–43^ Obviously, the specific surface area of the sample increases with the incorporation of Cu. The specific surface area of Cu1.5–PCN reaches 182.14 m^2^ g^−1^, which is 3.6 times larger than that of PCN (48.31 m^2^ g^−1^). The specific surface area of BCN is relatively smaller (19.79 m^2^ g^−1^). This will promote the separation and transfer of charge carriers and improve the performance of photocatalytic hydrogen evolution.

**Fig. 5:**
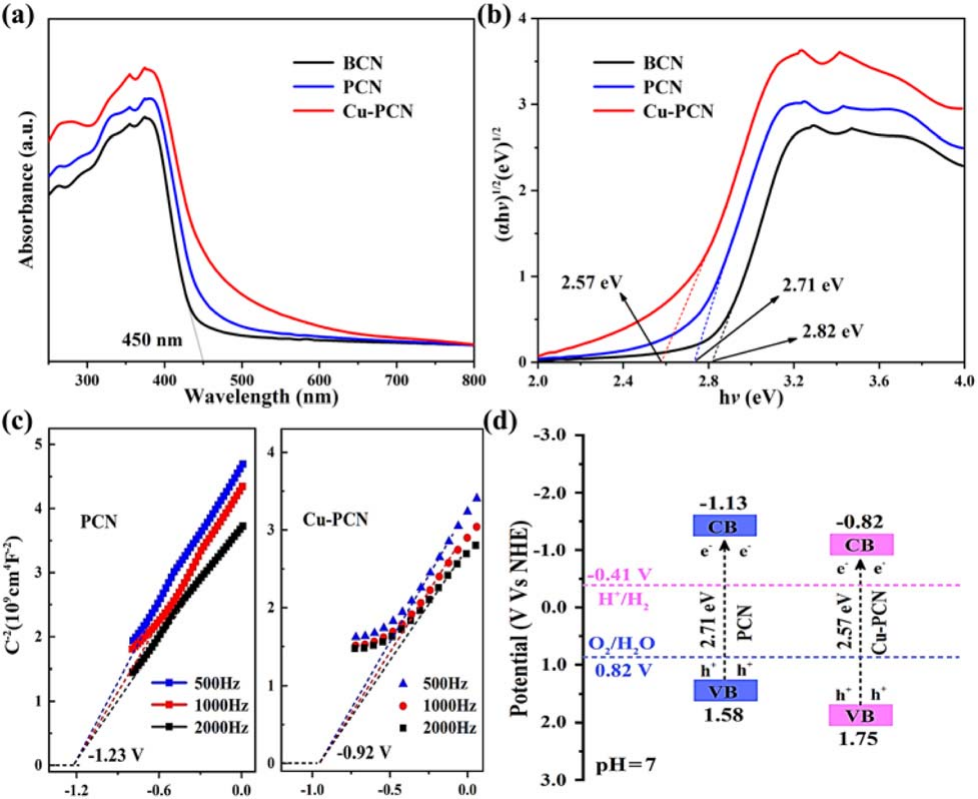
(a) UV-vis DRS spectrum and (b) plots of the (*αhν*)^1/2^*versus* the energy of absorbed light of the as-prepared samples, (c) Mott–Schottky plots of the PCN and Cu1.5–PCN under different frequency, (d) energy band structures of the samples.

In contrast to the BCN, the PCN samples display a significant redshift, and the light absorption band extends to the visible light region (380–700 nm), as shown in Fig. 5a. In addition, as the incorporation of Cu atoms, the visible light absorption threshold shifts to longer wavelengths, which indicates that the loading of Cu can affect the optical properties of carbon nitride polymers.^44,45^ Moreover, all photocatalysts have strong visible light absorption in the 280–460 wavelength range. The BCN reveals a distinct absorption band near 450 nm. The red shift and absorption increase is beneficial to improve the quantum efficiency and photocatalytic H_2_ evolution performance of Cu1.5–PCN. The optical band gaps of the Cu1.5–PCN samples are calculated using the Tauc plot, as shown in the Fig. 5b. The band gap (*E*_g_) of PCN is 2.71 eV, which is close to the previous report.^46,47^ After Cu incorporation, the band gap of the Cu1.5–PCN sample is reduced to 2.57 eV. The XPS valence band spectrum shows that the VB of PCN and Cu1.5–PCN are 1.58 and 1.75 eV, respectively (Fig. S10†). It has been confirmed that the introduction of heteroatoms in the carbon nitride polymers would reduce its band gap.^48,49^ It is noticeable that the color of the sample becomes darker with the increase of visible light absorption (Fig. S11†), extremely possible because of the superposition principle of Cu.^50^ These results demonstrate that the existence of Cu is beneficial in absorbing visible light.

The Mott–Schottky analysis is also used to investigate the energy band structure of the semiconductors. As displayed in Fig. 5c, it is obvious that both PCN and Cu1.5–PCN samples show a positive slope at different frequencies, indicating that they are n-type semiconductors. Compared with the Ag/AgCl (saturated KCl) reference electrode (pH = 7), the flat-band potential of the Cu1.5–PCN is −0.92 eV, which is equal to −0.72 V *vs.* the normal hydrogen electrode (NHE). In general, flat-band potential of an n-type semiconductor is probably 0.1 V lower than the CB, which is affected by the efficacious concentration of electrons and carrier.^51,52^ Consequently, the CB value of Cu1.5–PCN is −0.82 V. Correspondingly, the conduction band (CB) value of PCN is −1.13 V. If the conduction band potential (CB) is integrated into the *E*_g_ measured by the Tauc plot, it can be calculated using the formula below (1):

1 *E*_VB_ = *E*_g_ + *E*_CB_

The calculated conduction band potential (CB) of CN and Cu1.5–PCN samples is compatible with the potential obtained by Mott–Schottky analysis. Fig. 5d comprehensively shows the band structure and partial redox reaction potential of PCN and Cu1.5–PCN samples.^53^ It could be found that after introducing Cu, the band gap of the Cu1.5–PCN sample is narrowed. Although the conduction band position of Cu1.5–PCN moves down, it also meets the capacity necessary for the H_2_ production reaction. In short, the modification of these valence and conduction bands is thermodynamically suitable for water splitting to form hydrogen when exposed to sunlight.

**Fig. 6:**
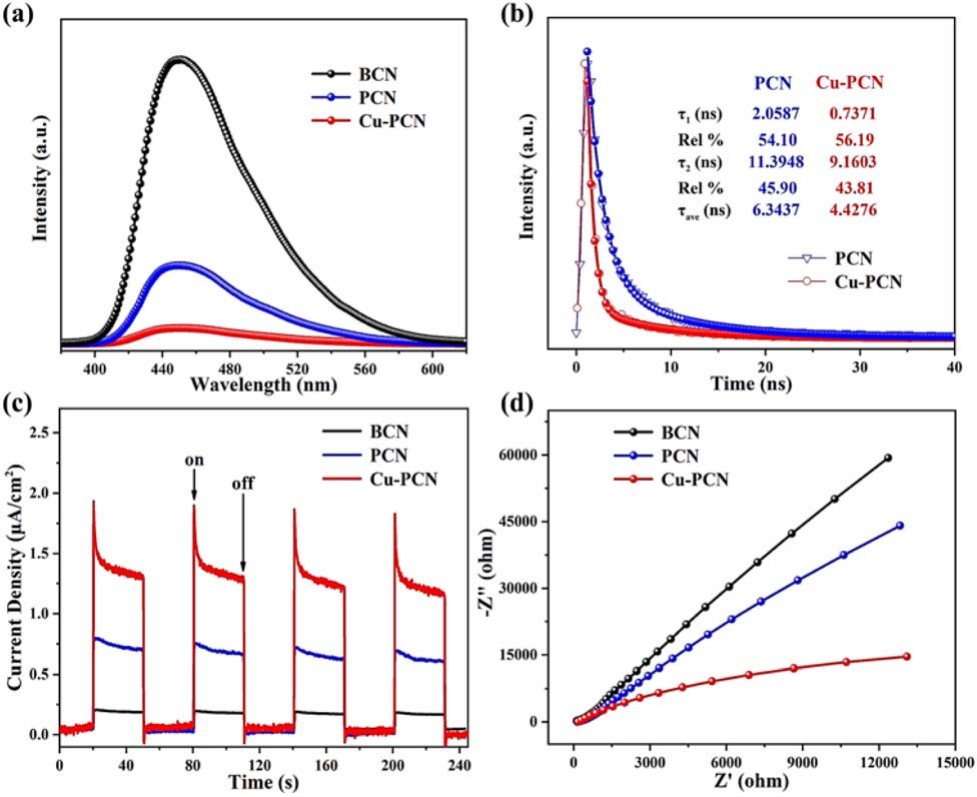
(a) The room temperature PL spectra for samples, (b) time-resolved PL decay spectrum for PCN and Cu1.5–PCN, (c) *I*–*t* curves and (d) EIS of the samples.

Generally, the rapid separation and effective transportation of photo-generated electron–hole pairs are considered to be the critical factor in the superior photocatalytic H_2_ generation performance. So the separation efficiency of carriers in the photocatalyst and the steady PL of the catalysts are investigated at room temperature, as seen in Fig. 6a. At a wavelength of about 450 nm, the BCN possess the strongest emission peak, which is accord with the photo-absorption results.^54–56^ Compared with the BCN, the prepared PCN photocatalysts display an obviously declined PL profile, which indicates that PCN supplies an abundant surface area to capture incident photons and provides efficient carrier separation. Particularly, among these samples, the PL intensity of Cu1.5–PCN is the lowest, indicating that Cu1.5–PCN can inhibit the recombination of the photo-excited carriers effectively. Time resolved photoluminescence spectrum (TRPL) is further carried out to understand the charge-carriers transfer dynamics (Fig. 6b). The carrier average lifetime (*τ*) of Cu1.5–PCN is 4.43 ns, lower than PCN (6.34 ns), indicating that the introduction of Cu could capture electrons and promote the separation of photo-generated charge carriers significantly, which is expected to have higher photocatalytic activity.^57,58^

Additionally, the separation and transportation behaviour of electron–hole pairs was also investigated through the transient photocurrent reactions for the prepared samples when exposed to visible light *via* some on–off cycles. It can be easily found that the Cu1.5–PCN possess the strongest photocurrent density in contrast to BCN and PCN photocatalysts, as shown in Fig. 6c, this suggests that the efficiency of photo-excited electron–hole pair migration and separation for Cu1.5–PCN are higher. The electrochemical impedance spectra (EIS) of samples were carried out, as seen in Fig. 6d. Generally, the smaller of arc radius for EIS, the smaller of resistance for electron migration, and the stronger of charge transfer ability.^59,60^ Compared with the other samples, the Nyquist curve of the Cu1.5–PCN has the smallest arc radius. Hence, the photo-excited electron–hole pairs transfer and migration efficiency of the Cu1.5–PCN sample is the highest, which is also in accordance with the results of PL analysis. Moreover, the hydrogen evolution polarization curves of the samples were investigated by the electrocatalytic dehydrogenation strategy as Fig. S12.† Distinctly, compared with BCN and PCN, Cu1.5–PCN has the lowest H_2_ evolution overpotential, which indicates that anchoring Cu into g-C_3_N_4_ can increase active sites and reduce the H_2_-reduction potential, thereby improving the surface reduction reaction kinetics of H_2_ evolution. Obviously, all the experimental results consistently show that when Cu atoms are anchored in the g-C_3_N_4_ cavity, numerous single-atom Cu active sites can improve the visible light trapping ability and separation of electron–hole pairs.

**Fig. 7:**
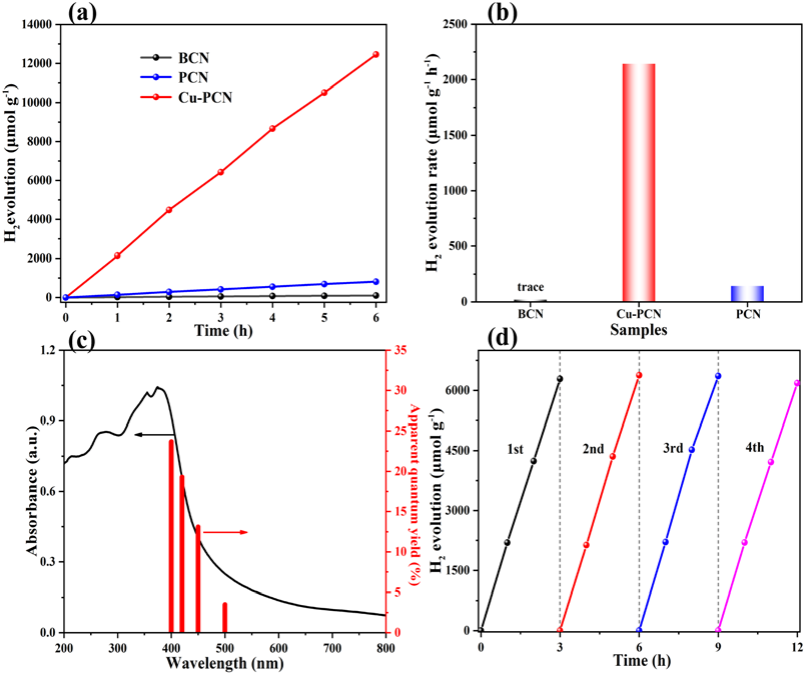
(a) The photocatalytic H_2_ evolution under visible light irradiation (*λ* > 420 nm) of samples, (b) comparison of photocatalytic HER rates of samples, (c) the AQYs of Cu1.5–PCN under different wavelengths, (d) photocatalytic H_2_ production stability test of Cu1.5–PCN at *λ* > 420 nm.

The photocatalytic H_2_ production activity of the synthesized catalysts has been evaluated in an aqueous solution containing TEOA under simulated sunlight irradiation (*λ* > 420 nm), without the additional noble metal cocatalyst. As shown in Fig. 7a, the H_2_ evolution activity of the BCN, PCN and Cu1.5–PCN samples were investigated. For all of these photocatalysts (Fig. 7a and S13a†), the amounts of H_2_ evolution were increased continuously with the prolonged irradiation time during experiments of 6 hours. The relevant contrast of the photocatalytic H_2_-evolution rate of the photocatalysts is displayed in Fig. 7b. The PCN sample shows medium photocatalytic hydrogen generation activity with a rate of 142.1 μmol h^−1^ g^−1^, which is almost 7 times higher than that of BCN (19.5 μmol h^−1^ g^−1^). The Cu1.5–PCN photocatalyst exhibits the highest H_2_ evolution rate of 2142.4 μmol h^−1^ g^−1^, which is far higher than that of PCN and BCN. The H_2_ generation rates of the other samples were 694.8, 1405.9 and 1017.4 μmol h^−1^ g^−1^, respectively (Fig. S13b†). According to Fig. 4, the specific surface area of Cu1.5–PCN, PCN and BCN is 182.14 m^2^ g^−1^, 48.31 m^2^ g^−1^ and 19.79 m^2^ g^−1^, respectively. After normalization, the hydrogen production rate of Cu1.5–PCN is still 4 times higher than PCN and almost 12 times of BCN. It confirms that the incorporation of single atomic Cu is beneficial to improve the catalytic performance of PCN. Notably, reducing the Cu content reduces the hydrogen production activity, indicating that Cu is the active site. In addition, excessive Cu also has an adverse effect on the catalytic performance due to the formation of CuO nanoparticles (Fig. S14†). The apparent quantum yields (AQYs) of Cu1.5–PCN (10 mg) for H_2_ evolution were evaluated for a three-hour reaction under the illumination of a monochromatic light at different wavelengths (Fig. 7c). The AQYs of Cu1.5–PCN at 400 nm, 420 nm, 450 nm and 500 nm are 23.7%, 19.3%, 13.6% and 3.5% respectively. Evidently, as the wavelength increasing, the apparent quantum yield gradually decreases, but there is still 3.5% at 500 nm, which indicates that the photocatalyst still has a better photocatalytic activity under visible light. Apart from excellent H_2_ production activity, the recycling and endurance of the photocatalysts are also one of the key elements influencing its practical application. Thus, the photo-stability of the Cu1.5–PCN catalyst was investigated by controlling irrelevant variables, under the same experimental conditions, through a cycling photocatalytic reaction of four rounds. Fig. 7d shows a 12 hour recycling tests of the Cu1.5–PCN sample under visible light irradiation. Even after four rounds of photocatalytic reactions within 12 hours, the H_2_ generation ability of the Cu1.5–PCN sample has insignificant changes, demonstrating its high photo-stability.

The comparison of the photocatalytic H_2_-evolution performance between the Cu1.5–PCN sample and the latest reported g-C_3_N_4_-based photocatalysts are presented in Table S2.† Obviously, the excellent photocatalytic H_2_ evolution performance of the Cu1.5–PCN is superior.

**Fig. 8:**
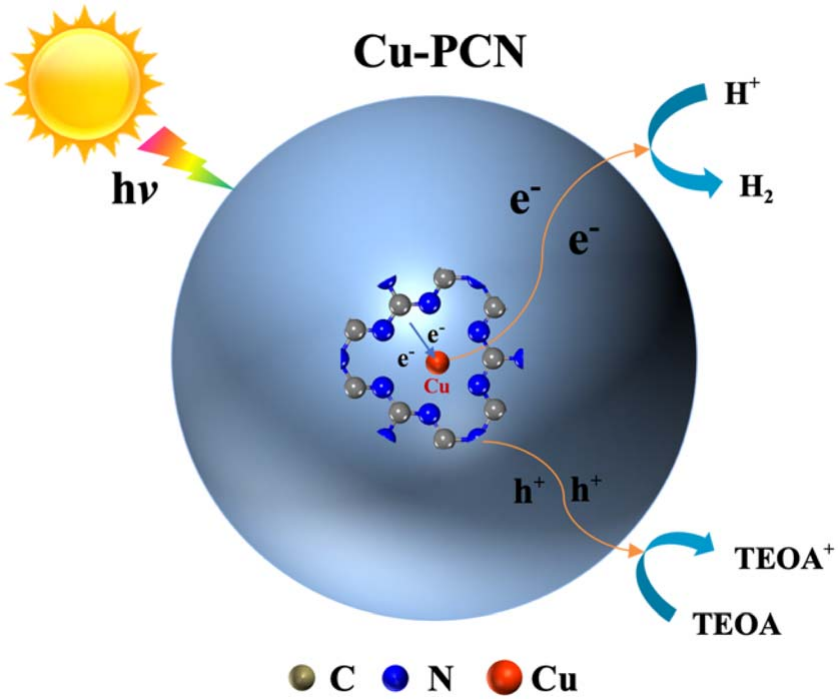
A proposed photocatalytic H_2_ production mechanism schematic of single atom Cu1.5–PCN photocatalytic systems.

Based on the above experimental and theoretical results, the monoatomic Cu anchored on porous g-C_3_N_4_ plays an important role in the process of photocatalytic hydrogen evolution. The reaction mechanism of the photocatalytic H_2_ production process for Cu1.5–PCN catalytic materials is shown in Fig. 8. The g-C_3_N_4_ nanosheets still retain the porous structure after the anchoring single atom Cu, this expands the region of contact between the photocatalysts and water, thereby helping to provide more reactive sites. At the same time, the energy barrier of water adsorption is reduced, making it easier for water molecules to be adsorbed by the photocatalysts. Under simulated solar light illumination, porous g-C_3_N_4_ generates electrons and holes in the CB and VB by absorbing photons.

Subsequently, owing to the electron transmission distance is shorten by the Cu–N bond in the Cu1.5–PCN, the photo-excited electrons are quickly transferred to the Cu atoms surface through the Cu–N charge bridge, and captured by the single atom Cu where water molecules are reduced to generate hydrogen. Meanwhile, the photo-generated holes in the valence band are removed by TEOA molecules. Significantly, the close electronic interaction between Cu and N makes single atom Cu anchored on carbon nitride in the form of Cu–N, enhancing the synergy between Cu and carbon nitride. In brief, as an electron trap and active site, Cu–N charge bridge effectively promotes the separation of photo-excited carriers, thereby greatly enhancing the photocatalytic activity of carbon nitride.

## 4 Conclusions

In summary, a single-atom Cu incorporated and porous g-C_3_N_4_ photocatalyst was successfully synthesized by a straightforward one-pot calcination method. By forming bonds with N (termed as charge bridge), the single Cu atom has been successfully anchored on porous g-C_3_N_4_ and stabilized in the form of Cu–N. The formed Cu–N charge bridge acts as the active sites for the adsorption of water molecules, hydrogen reduction and desorption, and also provides a fast electron transfer channel in order to separate charge carriers. The synthesized catalyst broadens the response range of visible light, increases the efficiency of electron–hole separation and transfer, and reduces the hydrogen evolution reaction energy barrier. As a result, the H_2_ evolution performance of the photocatalysts is markedly improved at visible light irradiation (*λ* > 420 nm). The highest H_2_ evolution rate reaches 2142.4 μmol h^−1^ g^−1^, and corresponding AQY is 19.3% (*λ* = 420 nm). The Cu1.5–PCN photocatalyst has potential industrial-scale applications in clean energy due to its low cost and robust performance and has great implications in the design of metallic single-atom photocatalysts for water splitting.

## Author contributions

B. Xiao and Q. Liu conceived and supervised the progress of the entire project. T. Zhou and H. Wei developed the original idea, designed the experiments and the DFT calculations, prepared the materials, performed and analyzed the characterizations of the materials. Y. Zhang, T. Lv, J. Lu and L. Duan helped with the interpretation of the results. T. Lv, J. Lu and J. Zhang helped with the characterization and corresponding analysis. All authors wrote the manuscript and have reviewed, discussed and approved the results and conclusions of this article.

## Conflicts of interest

There are no conflicts of interest to declare.

## Supplementary Material

RA-013-D3RA00775H-s001
