# Supplementary material for: Anchored Cu single atoms on porous g-C3N4 for superior photocatalytic H2 evolution from water splitting
Source: RSC Adv. 2023 Mar 17;13(13):8915–22. doi: 10.1039/d3ra00775h (PMC10020988; doi:10.1039/d3ra00775h)
Supplement: RA-013-D3RA00775H-s001 [file RA-013-D3RA00775H-s001.pdf]

Electronic Supplementary Material (ESI) for RSC Advances.
This journal is © The Royal Society of Chemistry 2023

Supporting Information

Anchored Cu Single Atoms on Porous g-C_3N_4 for Superior Photocatalytic H_2 Evolution from Water

Tong Zhou, # Haitang Wei, # Bin Xiao*, Tianping Lv, Liangfei Duan, Qingjie Lu, Jin Zhang, Yumin Zhang, Qingju Liu

National Center for International Research on Photoelectric and Energy Materials, Yunnan Key Laboratory for Micro/Nano Materials & Technology, School of Materials and Energy, Yunnan University, Kunming 650091, P. R. China.

# Equal contribution for this work.

* Corresponding author: Bin Xiao, *E-mail:* ynuxb2011@163.com (B. Xiao).

Experimental section

Photocatalytic hydrogen evolution

The photocatalytic H_2 evolution in pure water was performed in a closed Pyrex reactor and evacuation system (Perfect light, Beijing). The simulated light source is a Xeon-lamp (300 W), which equipped with a 420 nm cutoff filter. Typically, 10 mg of the prepared samples were dispersed in 80 mL of 15vol% triethanolamine under constant stirring (TEOA, a sacrificial agent for the photo-generated holes). The reaction temperature was kept at 281 K through the cooling water circulation system. Prior to each water splitting test, the reaction solution and reactor were degassed by a vacuum pump and N_2 to completely remove the air. The produced H_2 were detected by gas chromatograph (GC 9790) connected with a thermal conductivity detector (TCD). The apparent quantum yield (AQY) of the photocatalyst was evaluated by using a series of optical filters (400 nm, 420 nm, 450 nm and 500 nm). The AQY was measured through the following equation (1)[1,2]:

$$\text{AQY (\%)} = \frac{\text{number of reacted electrons}}{\text{number of incident photons}} \times 100\%$$

$$= \frac{2\text{M}N_A\text{hc}}{IAt\lambda} \times 100\% \qquad (1)$$

where M is the amount of hydrogen, N_A is the Avogadro's constant, h is the Planck constant, c is the light velocity, I is the intensity of the light, A is the irradiation area, t is the reaction time, and λ is the light wavelength.

Characterization

The crystalline structures of the prepared samples were characterized by powder X-ray diffraction (XRD, TTRIII-18KW, Japan) patterns with Cu-K_α radiation (λ = 0.1541 nm) operating at 40 kV and 40 mA. The surface morphology, size and lattice fringe measurements were obtained by field emission scanning electron microscope (Nova Nano SEM 450) and transmission electron microscope (JEM-2100, JEOL Ltd.), respectively. AC HAADF-STEM images and EDX elemental mappings were carried out by a Cs-corrected FEI Titan Themis3 G2 300 equipped with a Super-X EDS detector and operated at 300 kV. The X-ray photoelectron spectroscopy (XPS) analysis were recorded on an

ESCALAB 250 Xi (Thermo, USA) XPS instrument with Al Kα as the excitation source (hν = 1486.6 eV). The UV–vis diffuse reflectance spectra (DRS) of as-prepared samples were recorded on a Hitachi UV-3101 UV-Vis-near-IR spectrophotometer in the range of 320–800 nm with $BaSO_4$ as the reflectance standard. Fourier transform infrared spectroscopy (FT-IR) was performed on a Vector 33 infrared Fourier spectrometer. The photoluminescence (PL) emission spectra were measured on a fluorescence spectrophotometer (G9800A, Agilent Technologies). The TRPL spectroscopy was measured by a fluorescence lifetime spectrophotometer (FLS920, Edinburgh). The N_2 adsorption/desorption measurement was performed by the Micromeritcs ASAP 2010 analyzer and the Brunauer Emmett-Teller (BET) surface area was analyzed by BET theory.

Photoelectrochemical measurements

The working electrodes were prepared by the following procedure: 4 mg sample was dispersed in the mixed solvent containing 20 μL Nafion solution (5wt%), 200 μL ethanol and 800 μL deionized water. After the above mixture was ground and ultrasonically dispersed uniformly, a certain amount of slurry was spin-coated on the FTO conductive surface with an effective area of 1.5 cm^2, and dried at 60 °C for 5 h. The electrochemical measurements were measured using a CHI660E electrochemical working station with a conventional standard three-electrode setup in 0.5 M Na_2SO_4 aqueous solution. The Xenon lamp (300 W) was used as the simulated light source, which equipped with a cutoff filter ($\lambda >$ 420 nm). The linear sweep voltammetry (LSV) curves were detected from −1.5 - 1.5 V. The transient photocurrent response measurements were recorded with repeated on/off illumination from visible light. The Mott–Schottky plots were performed at -1.4 - 1.0 V with 500, 1000 and 2000 Hz. Electrochemical impedance spectroscopy (EIS) were detected at a 10 mV vs Ag/AgCl reference electrode of applied voltage with a three-electrode configuration over a frequency range from 10 kHz to 0.1 Hz.

DFT calculations

All calculations were performed using density functional theory (DFT) implemented in DMol3 package. The exchange correlation effects were accounted by the generalized gradient approximation (GGA) of Perdew-Burke-Ernzerhof (PBE). The functional type is GGA PBE. The electron-ion interaction was treated using effective core potential (ECP). The global orbital cutoff of 4.0 Å was

adopted to improve computational performance. The Brillouin zone is sampled by Monkhorst-Pack 3×3×1 k-point for all calculations. The semi-empirical London dispersion corrections of Grimme were conducted to calculate the interactions between absorbers and sample. The tolerances of the energy, gradient, and displacement convergence were 2×10^{-5} hartree, 4×10^{-3} hartree per Å, and 5×10^{-3}Å.

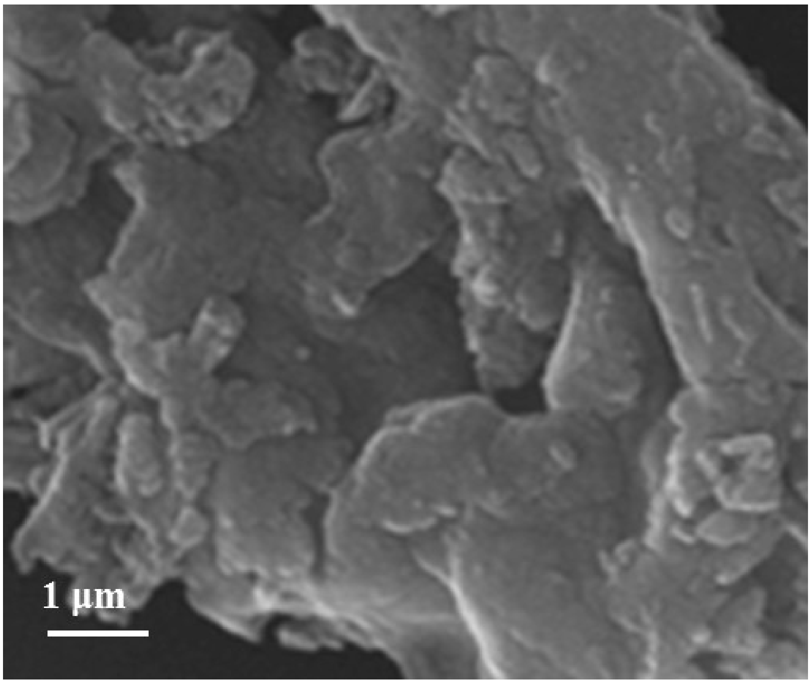

Figure S1. SEM image of the bulk g-C_3N_4 (BCN).

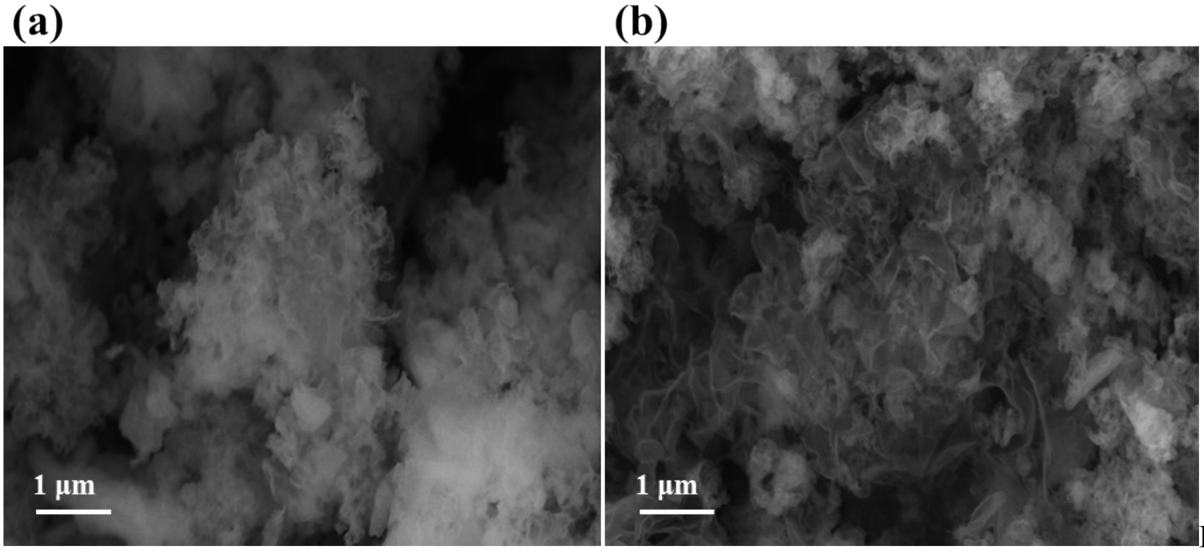

Figure S2. SEM images of (a) PCN and (b) Cu-PCN samples.

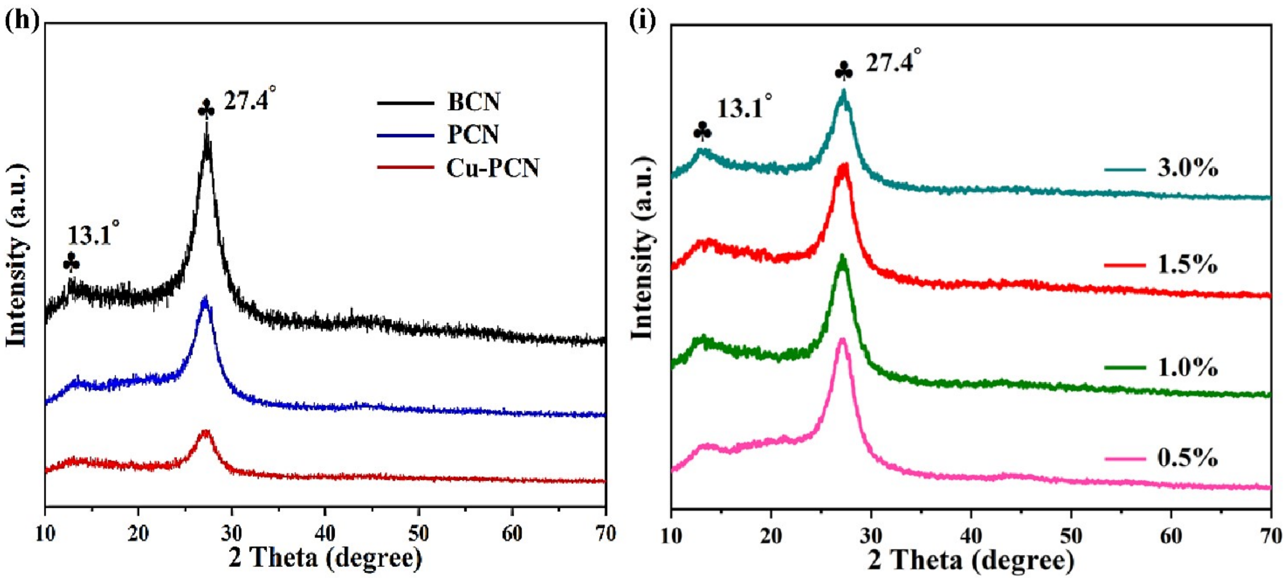

Figure S3. XRD patterns for BCN, PCN and the Cu-PCN with different Cu loading amount.

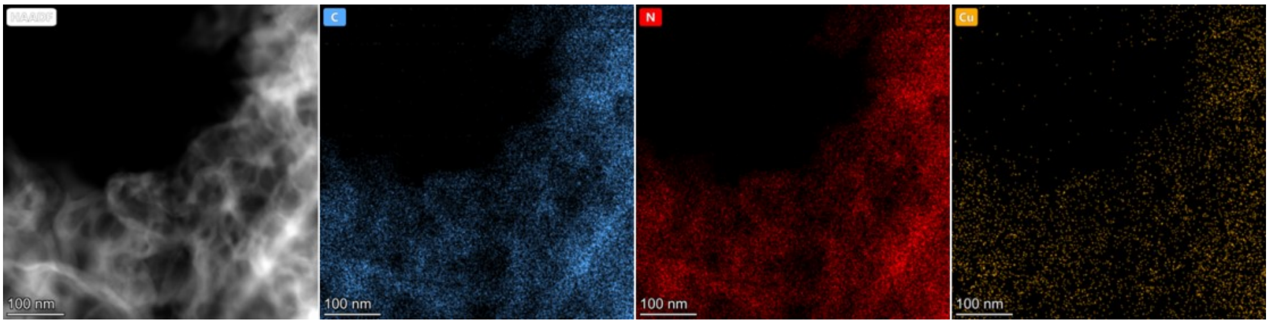

Figure S4. HAADF-STEM and EDS elemental mapping images of Cu-PCN.

(a)

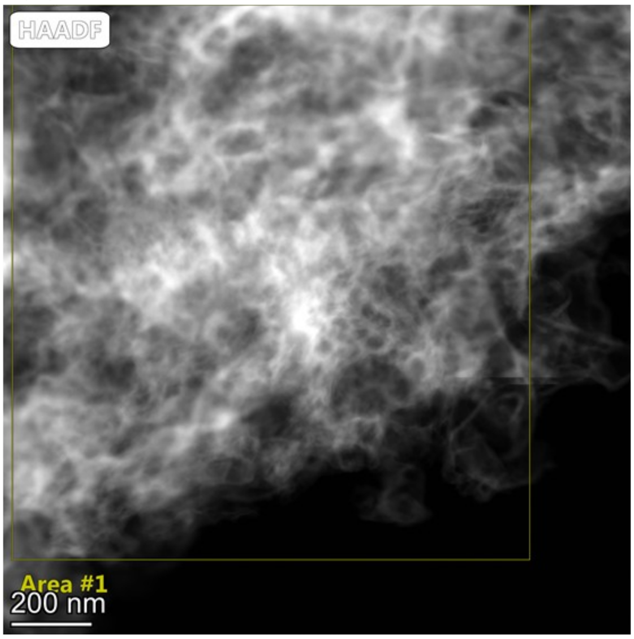

(b)

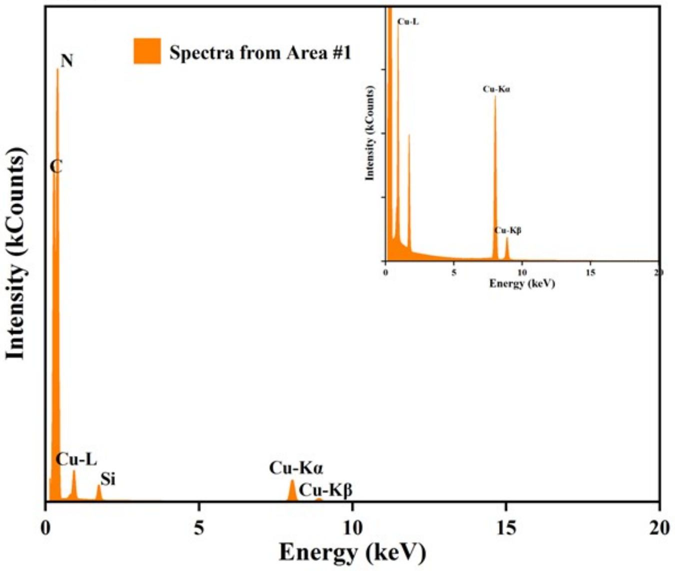

Figure S5. HR-TEM image of (a) Cu-PCN and (b) EDS spectrum indicating the absence of any contaminant element (Si matrix).

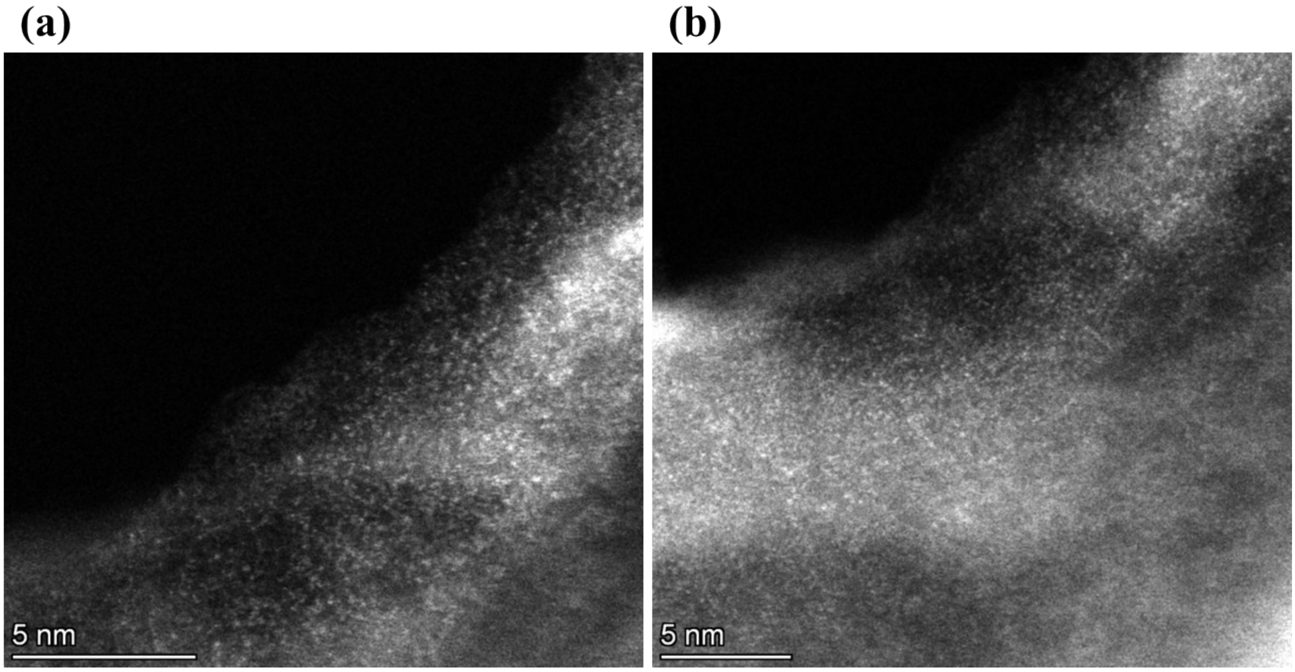

Figure S6. High–angle annular dark–field scanning transmission electron microscopy (HAADF–STEM) images of Cu-PCN samples.

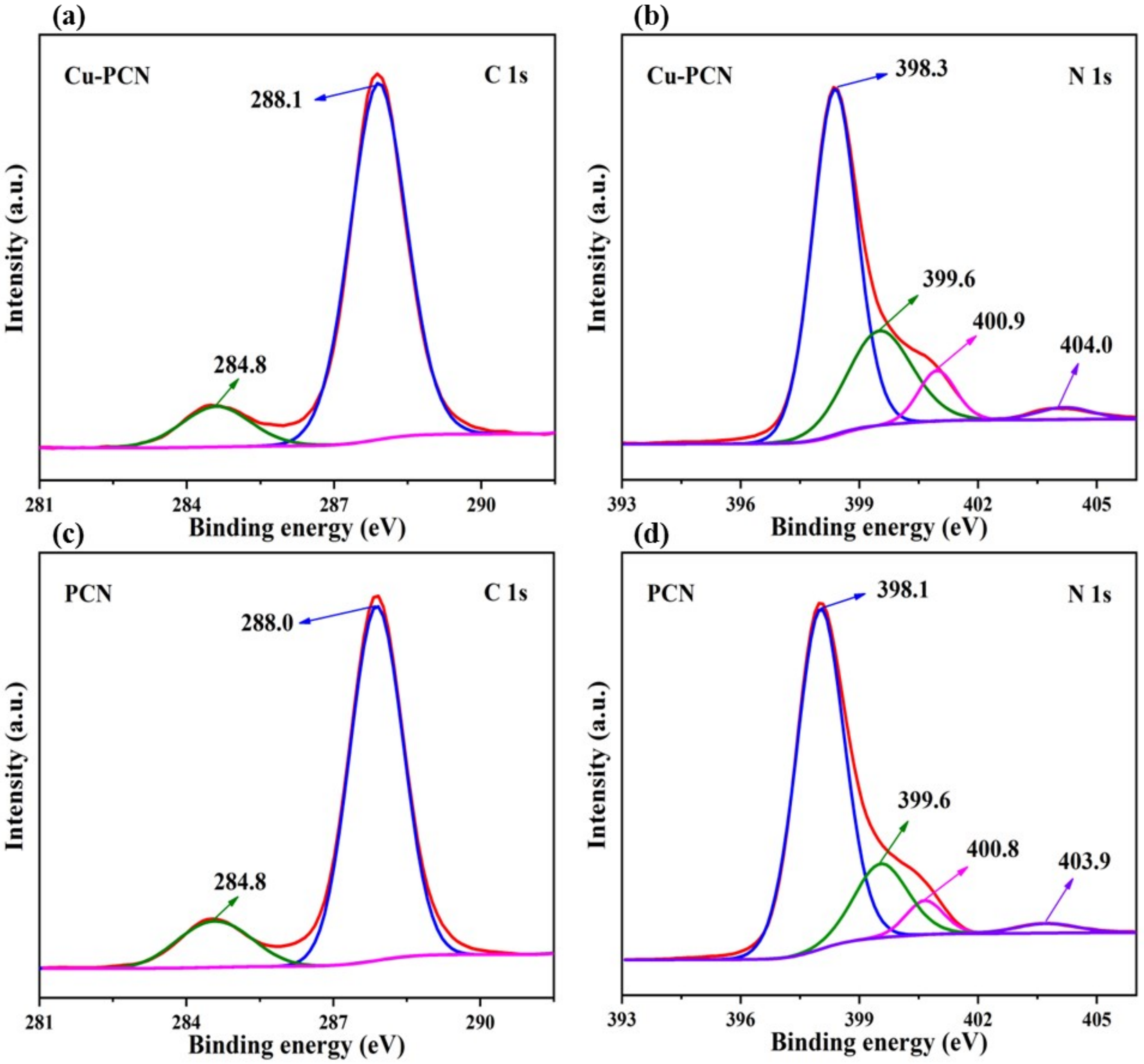

Figure S7. The XPS (a, c) C 1s and (b, d) N 1s spectra of Cu-PCN and PCN.

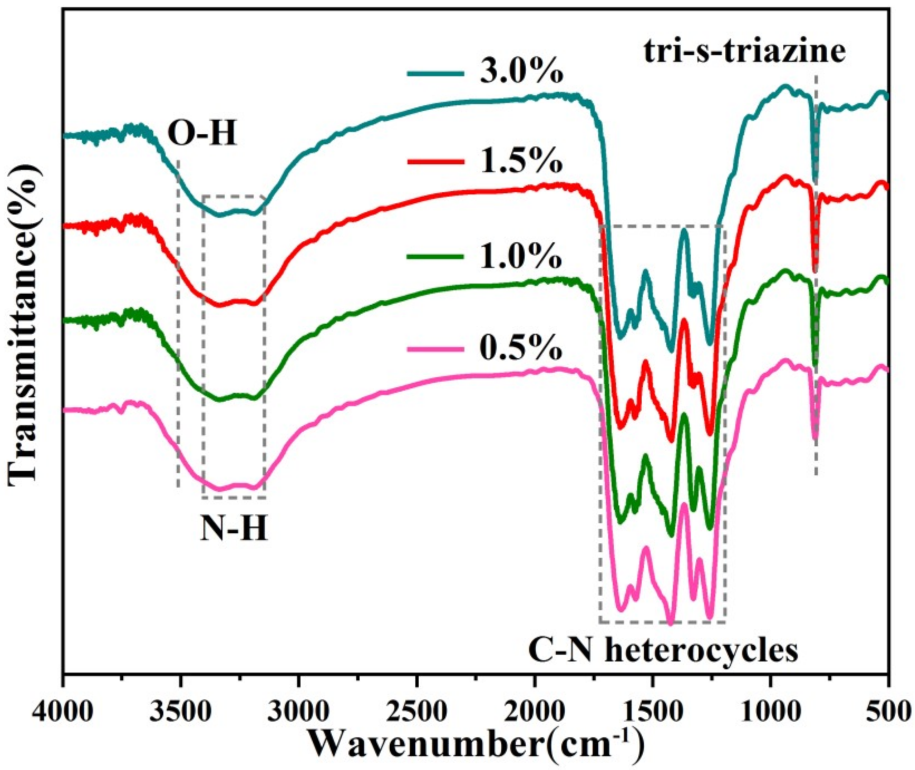

Figure S8. FT-IR spectra of the Cu-PCN with different Cu loading amount.

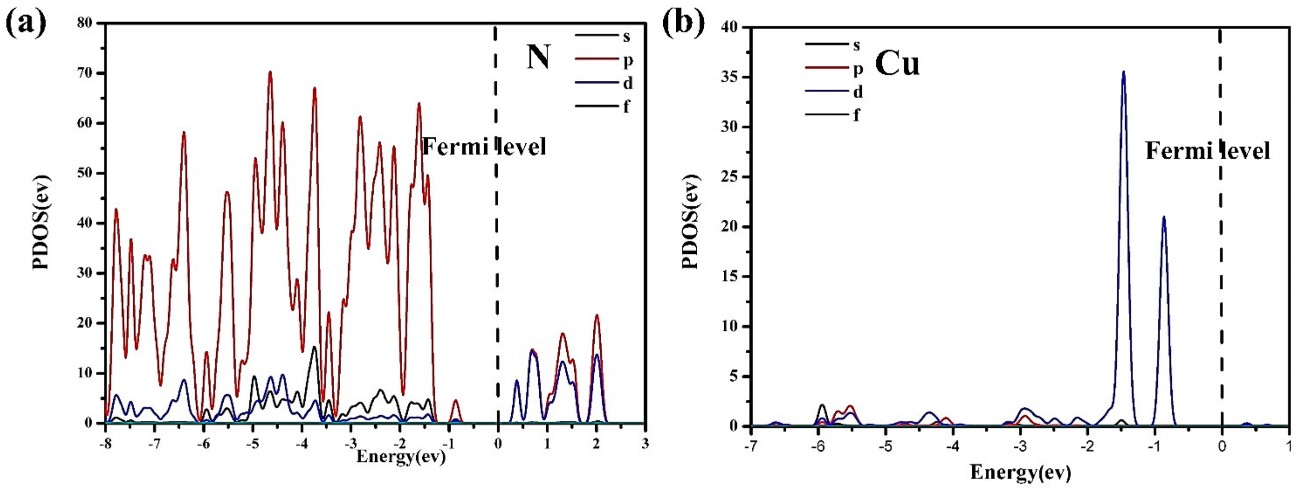

Figure S9 Calculated the projected DOSs for different orbitals on (a) N and (a) Cu elements.

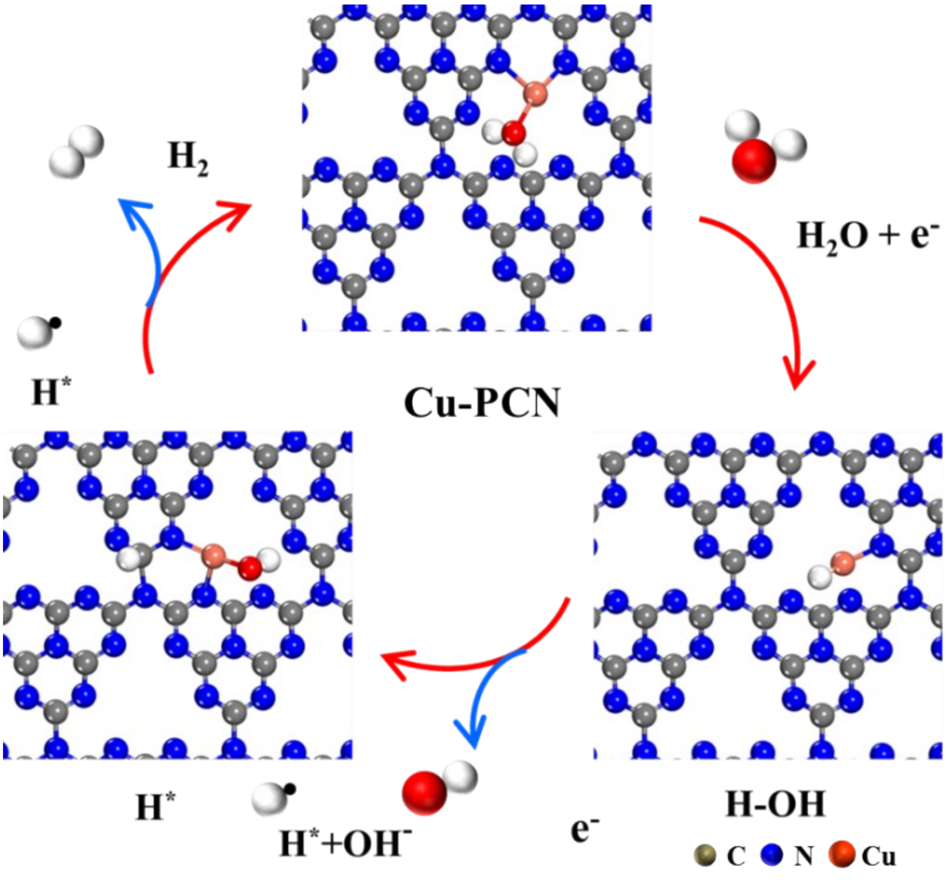

Figure S10. H_2 evolution process of Cu-PCN.

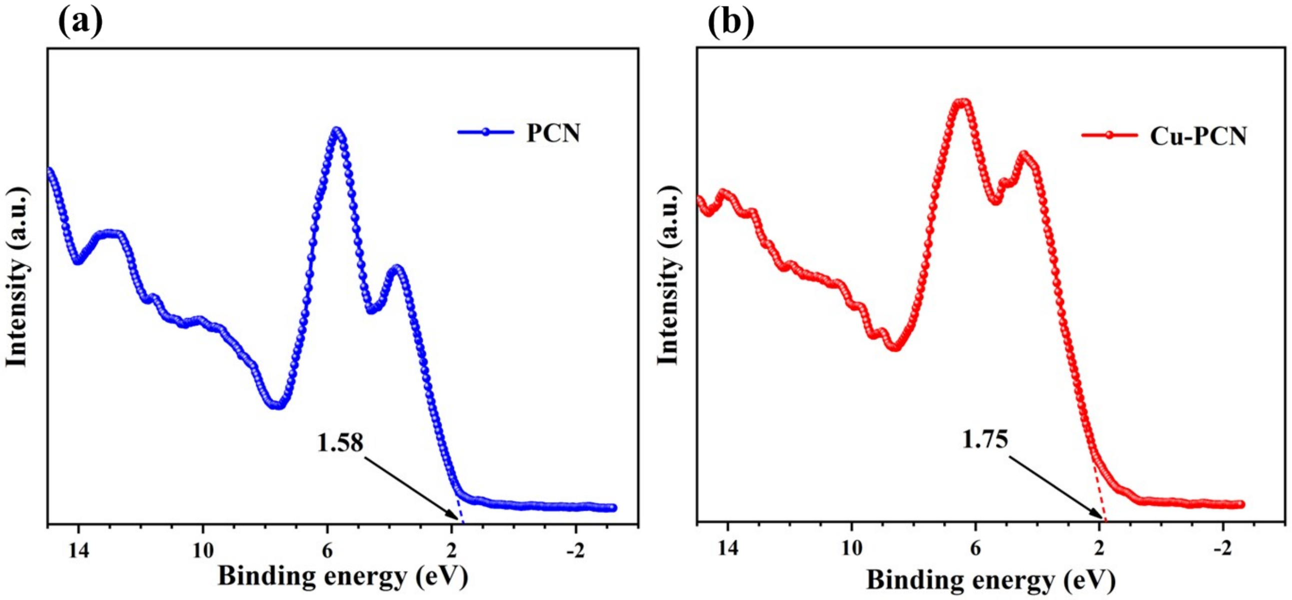

Figure S11. VB XPS spectra of (a) the pristine PCN and (b) Cu-PCN.

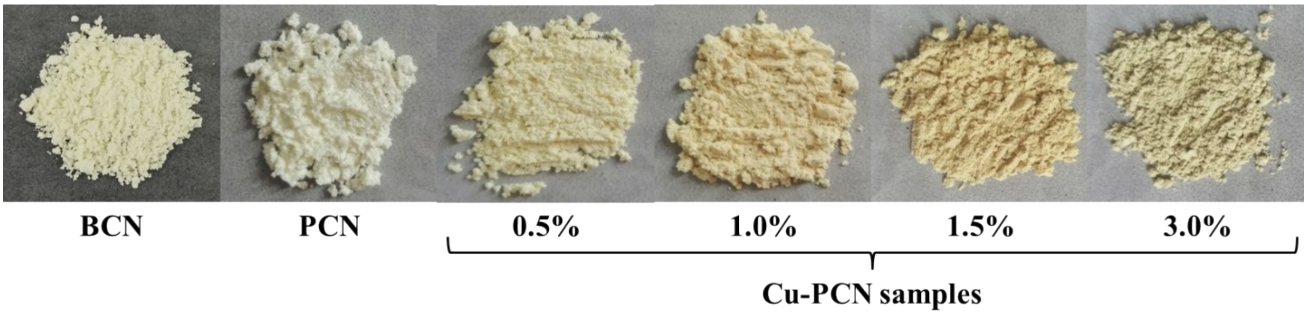

Figure S12. Optical photographs of g-C_3N_4 and Cu-PCN samples containing different amounts of Cu.

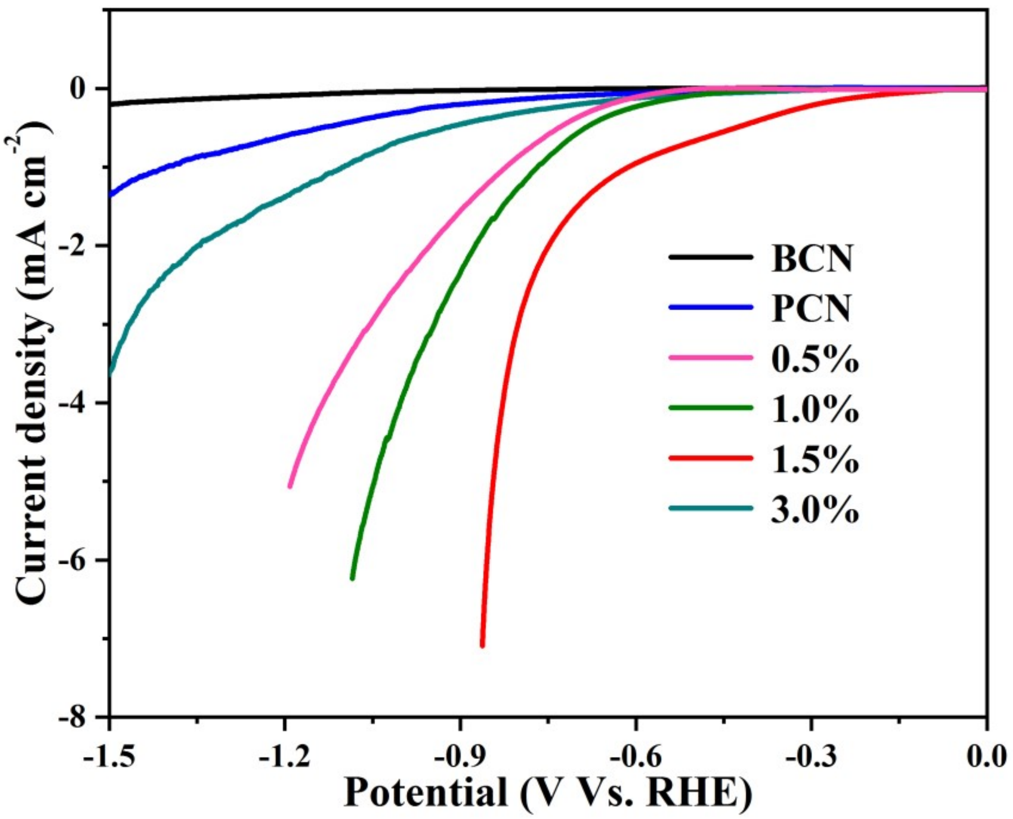

Figure S13. Linear sweep voltammetry curves of the prepared samples.

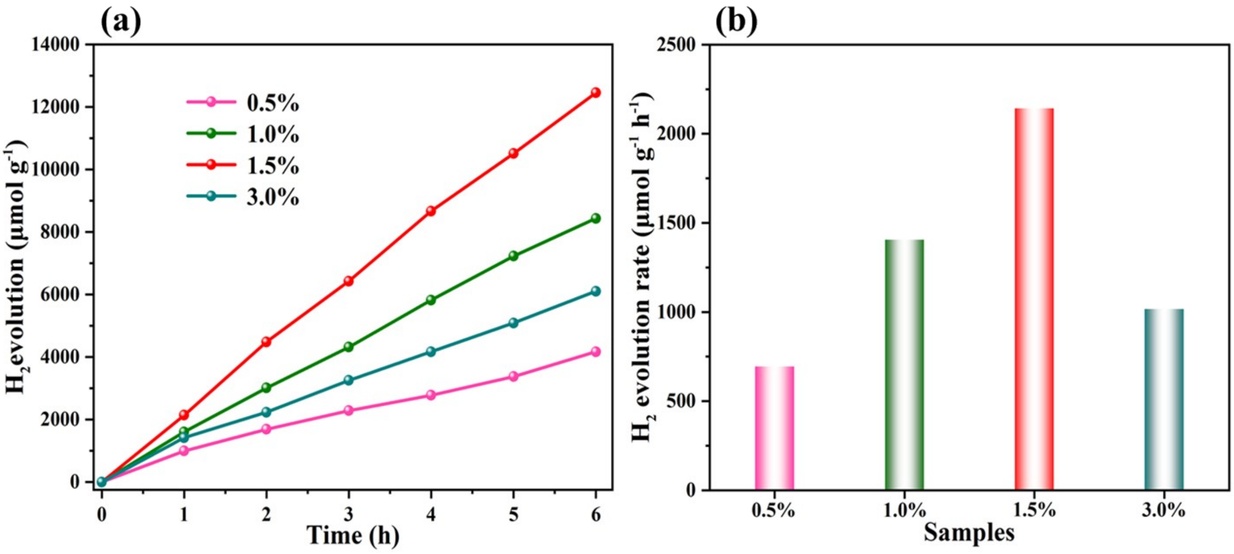

Figure S14. (a) Photocatalytic H_2 evolution and (b) comparison of rate of as-prepared catalysts.

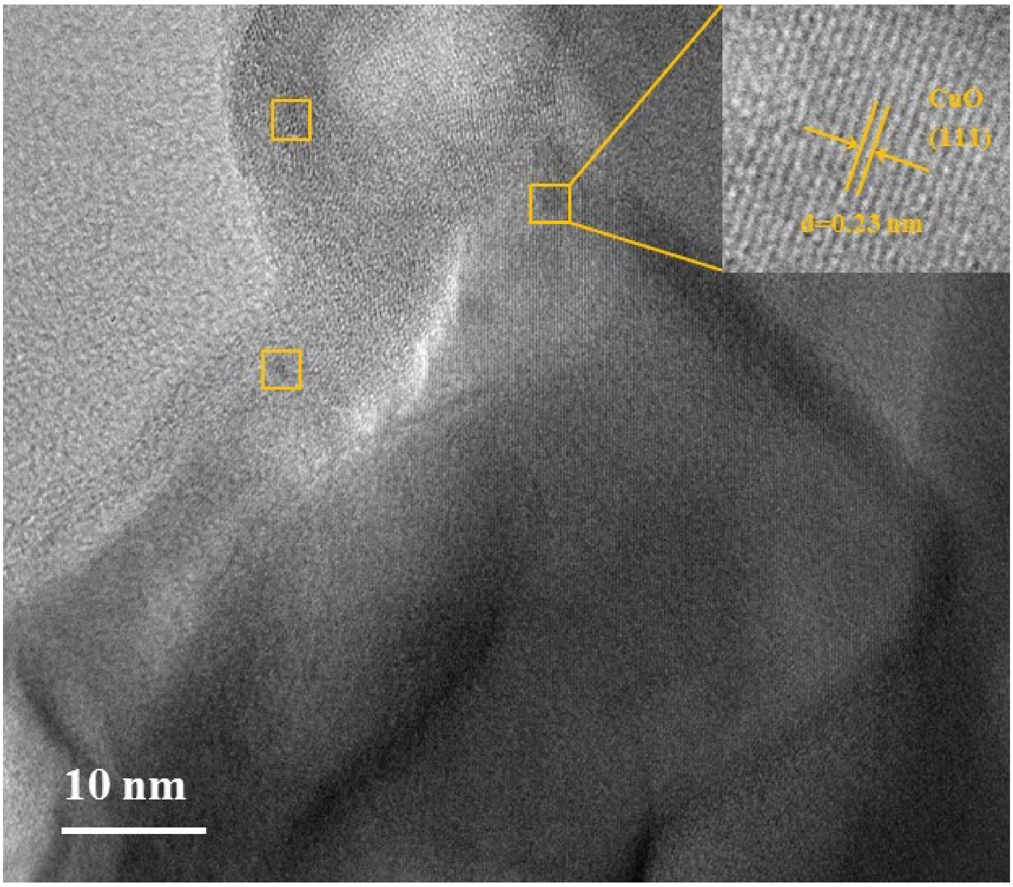

Figure S15. HR-TEM image of Cu-PCN (3.0wt% Cu) showing the formation of CuO Nanoparticles.

Table S1. Textural properties of the BCN, PCN and Cu-PCN samples.

Samples	S_{BET} (m^2 g^{-1})	Average pore size (nm)	Pore volume (cm^3 g^{-1})
**BCN**	19.79	---	---
**PCN**	48.31	23.79	0.36
**Cu-PCN**	182.14	20.67	0.29

Table S2. Comparison of H_2-evolution performance for Cu-PCN with the reported g-C_3N_4 photocatalysts and Cu atoms-based photocatalyst.

Photocatalysts	Cocatalyst	Light source	H_2-evolution rate (μmol h^{-1} g^{-1})	AQY	Ref.
WO_3/g-C_3N_4	2wt% Pt	300 W Xe lamp	1060	--	[3]
Ag@g-C_3N_4/CdS	--	λ > 420 nm	204.19	3.94% at 450nm	[4]
AgI/g-C_3N_4	0.6wt% Pt	λ > 420 nm	180	3.15% at 450nm	[5]
MoN nanosheets/g-C_3N_4	--	λ > 400 nm	240	0.09% at 400nm	[6]
Co/g-C_3N_4	--	λ > 420 nm	544.2	--	[7]
Ni_2P-P/g-C_3N_4	--	λ > 420 nm	1250	--	[8]
g-C_3N_4/$CoTiO_3$	2wt% Pt	λ > 400 nm	118	--	[9]
(SiC/C)/g-C_3N_4	1wt% Pt	λ > 420 nm	200.2	--	[10]
$K_2Ti_6O_{13}$ nanorod/ g-C_3N_4	Rh	λ > 420 nm	18.65	7.8% at 420nm	[11]
Cu-g-C3N4	--	λ > 420 nm	526	--	[12]
PdCu/g-C_3N4	--	λ > 420 nm	1075	--	[13]
**CuSA-g-C_3N_4**	**--**	**λ > 420 nm**	**2142.4**	**19.3% at 420nm**	**Our work**

Supplementary references

[1] J. Zhou, J. Zhao and R. Liu, *Appl. Catal. B: Environ.*, 2020, 278, 119265.

[2] M. Melchionna and P. Fornasiero, *ACS Catal.*, 2020, 10, 5493-5501.

[3] J. H. Won, M. K. Kim, H.-S. Oh and H. M. Jeong, Applied Surface Science, 2023, 612, 155838.

[4] Y. Shang, H. Fan, Y. Chen, W. Dong and W. Wang, Journal of Alloys and Compounds, 2023, 933, 167620.

[5] Y. Shang, H. Fan, X. Yang, W. Dong and W. Wang, Journal of colloid and interface science, 2023, 631, 269-280.

[6] J. Du, Y. Shen, F. Yang, J. Wei, K. Xu, X. Li and C. An, Applied Surface Science, 2023, 608, 155199.

[7] T. Song, X. Zhang, K. Matras-Postolek and P. Yang, Journal of Environmental Chemical Engineering, 2022, 10, 108747.

[8] J. Tang, X. Li, Y. Ma, N. Xu, Y. Liu and Q. Zhang, Applied Surface Science, 2022, 602, 154228.

[9] A. Meng, S. Zhou, D. Wen, P. Han and Y. Su, Chinese Journal of Catalysis, 2022, 43, 2548-2557.

[10] M. Ma, G. Chu, L. Qiu, B. Cao, P. Li, Y. Shen, X. Chen, H. Kita and S. Duo, Nanotechnology, 2022, 33, 405704.

[11] M. Chandra, U. Guharoy and D. Pradhan, Acs Applied Materials & Interfaces, 2022, 14, 22122-22137.

[12] X. Zhang, X. R. Zhang, P. Yang, H.-S. Chen and S. P. Jiang, Chemical Engineering Journal, 2022, 450, 138030.

[13] B. Shi, Y. Li, C. Chen, W. Zou, Q. Tong, S. Yang, C. Sun, H. Wan, C. Tang and L. Dong, Environmental Chemistry, 2021, 40, 3112-3121.
